# Re-Examining the Link Between Grandiose Narcissism, Self-Esteem and the Processing of Negative Status-Related Information: An Experimental Study of Self-Affirmation

**DOI:** 10.3390/bs16101736

**Published:** 2026-09-24

**Authors:** Mark Hardaker, Elias Tsakanikos

**Affiliations:** 1 Institute of Psychiatry, Psychology & Neuroscience, Kings’ College London, London SE5 8AF, UK; 2 Department of Psychology, University of Crete, 74150 Rethymno, Crete, Greece

**Keywords:** narcissism, grandiose narcissism, self-affirmation, self-esteem, masked priming, bifactorial self-esteem model, personality processes

## Abstract

Research on the relationship between grandiose narcissism and self-esteem (SE) remains inconclusive. This study examined the effects of self-affirmation on self-regulation and the role of different dimensions of SE. Self-esteem was assessed using a bifactorial model derived from the RSES, distinguishing between positive (self-competence) and negative (self-derogation) components. The Narcissistic Personality Inventory (NPI) was used to measure grandiose narcissistic traits, interpreted according to a three-factor model comprising “Power”, “Exhibitionism”, and “Special person”. A total of 180 undergraduate participants were randomly assigned to either a self-affirmation or control condition. Participants then completed a masked priming-based lexical decision task to assess reaction-time responses to status-related cues, interpreted as a behavioural indicator consistent with self-regulatory processing. Self-affirmation was associated with higher self-competence and lower self-derogation. A preliminary Condition × Special person interaction at the 149 ms SOA indicated that the association between Special person narcissism and RT differences was attenuated in the self-affirmation condition. The findings provide preliminary evidence consistent with the mask model, suggesting that self-affirmation may influence the processing of negative status-related cues among individuals differing in narcissistic traits. These findings suggest that self-affirmation may warrant further investigation as a mechanism for modifying self-evaluative processing associated with narcissistic traits. It also offers some explanation for previous conflicting evidence between SE and narcissism. Finally, the study provides inter alia for the first time an experimental validation of a bifactorial model of SE.

## 1. Introduction

Narcissism (we refer to grandiose narcissism in this study) is a relatively stable trait characterised by either high or unstable self-esteem with short-term fluctuations (see Kernis, 2005, for a review), a sense of exaggerated self-importance, a strong sense of entitlement and a tendency to exploit others, as well as hostility and aggression when challenged (see Kjærvik & Bushman, 2021; Miller et al., 2017). Narcissism is often measured with the Narcissistic Personality Inventory (NPI; Raskin & Terry, 1988) and self-esteem (SE) by the Rosenberg Self-Esteem Scale (RSES; Rosenberg, 1965). The preponderance of relevant research establishes an association between narcissism and the RSES. For example, a large meta-analysis found only a modest positive association between narcissism and self-esteem (k = 11, N = 2963, r = 0.29; Twenge & Campbell, 2001). Consistent with these findings, Hyatt et al. (2018) reported a similarly small effect (k = 11, N = 4711, r = 0.28). Such findings appear somewhat paradoxical, given that an inflated sense of self-importance is considered a core characteristic of narcissism. It is still unclear how the relationship between narcissism and SE combines to shape behaviour, but considerable research shows that global associations between narcissism and SE may be simplistic and belie a more complex personality organisation, as illustrated by the mask model (e.g., see Jordan et al., 2021). Understanding the complex relationship between narcissism and self-esteem (SE) is of considerable theoretical and clinical importance. While Narcissistic Personality Disorder is typically associated with grandiosity, individuals with this disorder also display a range of underlying vulnerabilities, including shame, heightened sensitivity to criticism, mistrust, and reactive depression (see e.g., Pledger et al., 2025).

Our previous research has experimentally tested and evaluated the mask model, which proposes that beneath narcissists’ grandiose exterior lies a vulnerable core characterised by a strong need for self-affirmation (Hardaker et al., 2019; Hardaker & Tsakanikos, 2021, 2022; Sedikides, 2021). The mask model suggests that narcissistic rage originates from internal processes intended to protect the self from feelings of both low SE and insecurity (Bushman et al., 2009; Morf & Rhodewalt, 2001; Zeigler-Hill & Besser, 2013). The mask model can be thought of as external high SE and internal low SE, but efforts to find evidence for this proposition have been problematic (mainly because of the difficulties in measuring constructs like fragile, discrepant and contingent SE), and the model does not separate the more complex constructs that underlie narcissism and SE (see Bosson et al., 2008; Kuchynka & Bosson, 2018; Teachman et al., 2019). Evidence for the mask model begins to emerge when narcissistic traits are observed under experimental manipulations involving negative status-related cues such as “worthless” (Hardaker et al., 2019). We use the term negative status-related cues to refer to evaluative words that convey devaluation or diminished personal worth and may therefore be relevant to perceptions of social standing. Distinguishing between different subscales of narcissism and operationalising unidimensional SE more precisely could lead to more tangible results (Kuchynka & Bosson, 2018). We have previously shown that one way to effectively measure protective internal processes that seem to underlie narcissistic self-regulation to negative status-related cues is by using sequential masked priming paradigms (see Hardaker et al., 2019; Hardaker & Tsakanikos, 2021). Previous research suggests that sequential masked priming paradigms (see Hardaker et al., 2019; Hardaker & Tsakanikos, 2021) can reliably index internal processes compatible with self-regulation (see Horvath & Morf, 2009). We first explain in more detail the subtleties of the measures we select to use here.

Grandiose narcissism is the more typical expression of narcissism frequently measured with the NPI (Raskin & Terry, 1988), although several more contemporary measures are in use (see Foster et al., 2018). The NPI demonstrates high levels of reliability and predictive validity, particularly when using its underlying dimensions. The complex and multidimensional nature of narcissism is better understood when measuring its fundamental factors (Ackerman et al., 2018; Cai & Luo, 2018; Hyatt et al., 2018; Schmalbach et al., 2020; Zeigler-Hill & Besser, 2013). For instance, the model proposed by Kubarych et al. (2004) recommends that three discrete, correlated factors relating to “power”, “exhibitionism”, and being a “special person” underlie the NPI. Although their research was exploratory in nature, the model correlated well with the NEO Five-Factor Inventory (NEO-FFI, Costa & McCrae, 1992) and factors proposed by Corry et al. (2008). Similarly, SE is frequently measured by the one-dimensional RSES. “Previous research has generally examined the narcissism–self-esteem association using global RSES scores. This approach may obscure potentially different associations between narcissistic traits and positive versus negative self-evaluative content. Although bifactorial models of the RSES have been proposed, their application to narcissistic self-processing following self-affirmation remains limited. The present study therefore examines whether distinguishing self-competence from self-derogation provides additional information about the association between grandiose narcissism and responses to negative status-related cues.” We submit that more precise investigations that differentiate the fundamental components of narcissism and their associations with SE may provide more comprehensive results (e.g., see Zeigler-Hill & Vrabel, 2023; Zeigler-Hill & Besser, 2013).

The majority of research uses the unidimensional Rosenberg Self-Esteem Scale (RSES) to measure self-esteem. Despite extensive investigation of associations between NPI narcissism dimensions and total RSES scores (e.g., Hart et al., 2021; Ozimek et al., 2021; Xu et al., 2020), the potential utility of RSES subscales has received little attention, possibly because of the scale’s simplicity (McKay et al., 2014). Research investigating the dimensionality of the RSES has produced mixed results, with arguments about method effects due to inflated correlations, tests of model fit, reliability estimates and item loadings, but some research supports a bifactorial model of some description (Alessandri et al., 2015; McKay et al., 2014; Marsh et al., 2010; C. G. Richardson et al., 2009; Supple et al., 2013). Alessandri et al. (2015) and Supple et al. (2013) show that the RSES measures several related but distinct dimensions of SE. Alessandri et al. (2015) recommend two robust latent constructs reflected in the positive and negatively worded items on the RSES: positive items linked with self-competence (e.g., “I am able to do things as well as most other people”) and negative items linked with self-derogation (e.g., “I feel I do not have much to be proud of”). Data from tests of narcissistic hypervigilance to negative status-related cues show that self-affirmations reduce narcissistic self-regulation, but that self-affirmation shows no effect on SE (Hardaker & Tsakanikos, 2021). However, in contrast to our previous research, K. Richardson et al. (2021) and Zhu and Yzer (2021) show that the effect of self-affirmation is moderated by self-esteem. Given this discrepancy, we wondered whether using a bidimensional conceptualisation of self-esteem would reveal an effect on reaction time to negative cues, a relationship that has yet to be explored. Specifically, we considered whether using the bidimensional framework of self-esteem proposed by Alessandri et al. (2015) might provide a more nuanced understanding of narcissists’ responses to negative status-related cues after self-affirmation, potentially uncovering a previously undefined role of self-esteem in moderating these effects. We argue that applying Kubarych et al.’s (2004) three-factor model of narcissism, derived from the NPI, alongside Alessandri et al.’s (2015) two-factor model of self-esteem, derived from the RSES, within a self-affirmation paradigm will allow for a more nuanced investigation of narcissistic behaviour, particularly self-regulation (Hardaker & Tsakanikos, 2021). Such processes are closely associated with narcissistic vulnerability and the mask model of narcissism (Hardaker et al., 2019; Morf & Rhodewalt, 2001; Zeigler-Hill & Besser, 2013).

The present study integrates these perspectives by proposing that individual differences in grandiose narcissism may be associated with the processing of negative status-related information, and that this association may vary as a function of individuals’ self-evaluative profiles. Self-affirmation provides an experimental manipulation through which this proposed relationship can be examined. Specifically, if heightened processing of negative status-related information reflects self-protective regulation, affirming an alternative source of self-worth should attenuate the association between narcissistic traits and heightened processing of negative status-related information.

The main aim of this investigation was to examine whether self-affirmation influences self-regulation and whether these effects differ across the three dimensions of grandiose narcissism. Based on previous research (Kubarych et al., 2004), we hypothesised that self-affirmation attenuates self-regulation associated with the Power, Exhibitionism, and Special person dimensions of grandiose narcissism, as assessed by the NPI, although the magnitude of these effects may vary across dimensions. RT responses to negative status-related cues were used as a behavioural outcome relevant to the study’s theoretical account of self-regulatory processing, while recognising that RTs do not directly measure self-regulation:

**H1.**  *Participants in the self-affirmation condition will show higher self-competence and lower self-derogation than participants in the control condition*.

A second aim was to investigate the effects of self-affirmation on SE with particular attention to the distinction between self-competence and self-derogation proposed in bifactor models of SE (Alessandri et al., 2015; Supple et al., 2013). We hypothesised that self-affirmation would be associated with higher levels of self-competence and lower levels of self-derogation:

**H2.**  *The association between grandiose narcissism dimensions and reaction-time responses to negative status-related cues will differ between the self-affirmation and control conditions*.

Finally, we explored whether individual differences in narcissism and self-esteem were associated with RT responses to negative status-related cues. Specifically, we examined exploratory associations between the three narcissism dimensions, self-competence, self-derogation, and RT differences following negative primes. Because these analyses were not designed as direct tests of self-esteem effects on RT responses, they are interpreted as exploratory.

## 2. Method

### 2.1. Design

Participants were randomly assigned to the self-affirmation or control group. In each group, we tested the effect of stimulus onset asynchrony and narcissism on reaction time to status-related cues (dependent variable), as they are associated with specific self-esteem constructs. Participants were asked to complete a self-affirmation paradigm or an E-Prime-based lexical decision task and measures of narcissism and self-esteem. This research was approved by the University of Roehampton Ethics Committee.

### 2.2. Participants

A total of 180 participants (163 women) aged between 18 and 48 years (*M* = 20.68, *SD* = 4.57), who were all undergraduate students, were recruited from the University of Roehampton. All participants provided informed consent by completing a written consent form before taking part in this research. All participants had normal or corrected-to-normal vision. We randomly assigned participants to one of two conditions: affirmed or the non-affirmed (neutral) control. Each condition’s sample size was determined from comparable previous research (see Horvath & Morf, 2009; Krusemark et al., 2015), which reports typical effect sizes of η^2^_p_ = 0.14 (N = 64) and η^2^_p_ = 0.15 (N = 77) respectively. Using these as a guide, we conducted a G*Power 3.1 (Faul et al., 2009) a priori power analysis (*f*^2^ = 0.15; a = 0.05; *β* = 0.85; 2 predictors; affirmed, un-affirmed), which determined a minimum sample size of 82 participants for each condition. Participants were probed for suspicion after the priming task. Although the masking parameters were selected to minimise conscious awareness of the primes, no objective measure of prime awareness was administered. Consequently, the absence of conscious awareness of the primes cannot be empirically confirmed, and it remains possible that some participants detected the primes at above-chance levels.

### 2.3. Measures

Grandiose narcissism was measured with the 40-item forced-choice Narcissistic Personality Inventory (NPI; Raskin & Terry, 1988), where participants choose between two statements, such as A: “I like to look at myself in the mirror”, B: “I am not particularly interested in looking at myself in the mirror”; A: “I am no better or worse than most people”, B: “I think I am a special person” (*α* = 0.89).

Kubarych et al. (2004) show that the NPI comprises three highly correlated factors, suggesting a hierarchical structure in which a general narcissism factor sits at the top and divides into three interrelated domains—Power (e.g., “I have a strong will to power, I see myself as a good leader”: 15 items: 1, 5, 6, 8, 10, 11, 12, 16, 21, 24, 25, 27, 32, 33, 36) (*α* = 0.54), Exhibitionism (e.g., “I will usually show off if I get the chance”: 9 items: 2, 7, 18, 20, 26, 28, 30, 37, 38) (*α* = 0.61), and being a Special person (e.g., “I am more capable than other people”. 12 items: 3, 4, 9, 14, 17, 19, 23, 31, 34, 35, 39, 40), (*α* = 0.37)—(note: dimension-specific analyses are interpreted cautiously, and results involving Special person are preliminary).

Self-esteem was measured with the extensively used RSES (Rosenberg, 1965), where participants rate items such as “on the whole, I am satisfied with myself and at times, I think I am no good at all” on a scale from 1 (strongly disagree) to 5 (strongly agree) (*α* = 0.91).

Alessandri et al. (2015) tested a bifactor model of the RSES, in which all items load onto a general self-esteem factor (GSE), while positively worded items (positive self-competence; e.g., “On the whole, I am satisfied with myself, I feel that I have a number of good qualities”) and negatively worded items (self-derogation; e.g., “At times I think I am no good at all, I feel I do not have much to be proud of”) form two specific factors; Positive SE (self-competence) (α = 0.80) and Negative SE (self-derogation) (α = 0.80). Positive SE and Negative SE scores were calculated by summing the respective items for each subscale. Higher Positive SE scores indicate greater self-competence, whereas higher Negative SE scores indicate greater self-derogation. This structure separates global self-esteem from systematic variance due to item valence, yielding better model fit than unidimensional or correlated two-factor models.

### 2.4. Stimuli Apparatus and Procedure

Participants were individually tested in the cognitive laboratory in a small, enclosed cubicle. The cubicle contained all the test materials and a 21-inch CRT monitor set at an 85 Hertz refresh rate. Participants were first invited to complete the self-affirmation manipulation.

#### 2.4.1. Self-Affirmation Paradigm

To achieve our goal of priming self-affirmation without awareness, the following exercise is described as a “language exercise”. Initially participants are asked to complete a Preselection Sheet^1^, which requires them to read descriptions of five categories of topics (arts; politics; religion; social values; science) and then score them from 1–7 based on the degree to which that category is or is not personally important to them. Once participants have given a response to all five categories presented to them, they are asked to complete a Scrambled Sentence Exercise. There are six different Scrambled Sentence Exercises, one for each of the five value categories on the Preselection Sheet, and one neutral exercise for controls. Each participant’s Scrambled Sentence Exercise is selected based on the highest scoring value category chosen on the Preselection Sheet (or neutral for un-affirmed participants).

The Scrambled Sentence Exercise requires participants to unscramble four words to form short sentences, 15 of which are designed to implicitly reinforce the highest personal value category chosen by the participant in their Preselection Sheet. After completing the sentence unscrambling task, participants immediately began the lexical decision task.

#### 2.4.2. Lexical Decision Task

This task is used to detect the speed of participants’ responses to negative status-related cues. Faster responses were interpreted as an index of differential responding to negative status-related cues; the task does not directly measure vigilance or self-regulation (see Horvath & Morf, 2009).

As with previous experiments, this task followed the Morf and Rhodewalt (2001) procedure. Participants were asked to decide if a string of letters presented on screen was a word or non-word. Each trial started with the presentation of a small fixation cross, which remained on the screen for 505 ms. This was immediately followed by the first mask (KQHYTPDQFPBYL) displayed for 153 ms. Subsequently, one of the two masked prime words (negative or neutral) was displayed for 35 ms, and finally the second mask (FYVDLTMHQWSPW) was displayed for 24 ms. We implemented these masking procedures to prevent participants from becoming aware of the prime word (Wentura & Degner, 2010).

Following the masking procedure, a blank screen was displayed for either 90 ms or 176 ms (making a total stimulus onset asynchrony, SOA, of either 149 ms or 235 ms), and then the letter stimuli were immediately presented and remained on the screen until the participants reacted by pressing the appropriate button on a response box. Participants were given a response window of 1500 ms and were asked to react as speedily and accurately as comfortably possible.

After short verbal instructions and a one-minute practice trial, we asked participants to complete the lexical decision task, which consisted of 384 pseudo-randomised test trials divided into two blocks. Each trial comprised a randomised combination of one of the neutral/negative prime words followed by one of the 16 negative cues, 16 negative filler words, 16 neutral words or 48 non-words (see Note 1), which were presented at both the fast (149) and slow (235) stimulus onset asynchrony (SOA). The two SOAs were included to examine whether associations with negative status-related information varied as a function of the temporal interval between the masked prime and the target. The shorter SOA was intended to capture relatively early processing, whereas the longer SOA permitted examination of effects emerging at a relatively later stage. These interpretations should be understood as temporal distinctions in processing rather than as direct measures of distinct cognitive mechanisms. The lexical decision task required approximately 30 min to complete; we acknowledge that fatigue may be a possible source of variability.

When participants had finished the lexical decision task, they completed the self-report measures (the NPI and the RSES) and were thanked for their participation.

## 3. Results

### 3.1. Preliminary Analyses and Condition Differences in Self-Esteem Dimensions

A total of 180 undergraduate participants (163 women; age *M* = 20.68, *SD* = 4.57) were randomly assigned to the self-affirmation (*n* = 90) or control (*n* = 90) condition. Incorrect lexical decision responses and reaction times exceeding 1500 ms were excluded prior to analysis. No participants were excluded from the analyses.

To assess whether the self-affirmation manipulation influenced self-evaluative content as intended, the two self-esteem dimensions were compared between conditions. Participants in the self-affirmation condition reported higher self-competence (*M* = 10.80, *SD* = 2.37) than participants in the control condition (*M* = 8.68, *SD* = 3.34, *t*(178) = −4.76, *p* < 0.001, *d* = 0.71). They also reported lower self-derogation (*M* = 8.86, *SD* = 3.17) than participants in the control condition (*M* = 10.46, *SD* = 2.68, *t*(178) = 3.75, *p* < 0.001, *d* = 0.56). A multivariate test of the two self-esteem dimensions indicated a significant overall effect of condition (*F*(2, 177) = 14.10, *p* < 0.001, η^2^_p_ = 0.14). These findings supported H1, indicating that self-affirmation increased self-competence and reduced self-derogation relative to the control condition.

### 3.2. Primary Hypothesis-Driven Analyses of Narcissism × Affirmation Effects on Reaction Times (H2)

We proceeded to calculate difference scores, following the exclusion of wrong answers. For each SOA, we subtracted mean reaction times to negative cues on neutral-prime trials from mean reaction times to negative cues on negative-prime trials. Thus, negative scores reflect faster responding as a function of the negative prime, whereas positive scores reflect slower responding as a function of the negative prime, relative to the neutral prime. These hypothesis-driven analyses are not described as confirmatory because they used previously published data and were not preregistered.

To test H2, hierarchical regression analyses examined whether the associations between narcissism dimensions and RT difference scores differed between the self-affirmation and control conditions. Condition (0 = control, 1 = self-affirmation), centred narcissism dimension scores, and their interaction were entered as predictors. Condition and the relevant narcissism dimension were entered at Step 1, followed by the Condition × Narcissism interaction at Step 2.

#### 3.2.1. Special Narcissism and RT Differences at 149 ms SOA

The first model focused on the Special person dimension at the shorter SOA (149 ms), where previous research indicated the strongest effects. The Condition × Special interaction was significant (*B* = 7.80, *SE* = 3.60, *t*(176) = 2.17, *p* = 0.032, 95% CI [0.70, 14.90], Δ*R*^2^ = 0.03, Δ*F*(1, 176) = 4.71, *p* = 0.032).

Simple-slopes analyses indicated that, in the control condition, higher Special scores were associated with more negative RT difference scores, reflecting faster responses to negative cues following negative primes (*b* = −8.90, *SE* = 3.90, *t*(176) = −2.28, *p* = 0.024). In the self-affirmation condition, this association was substantially weaker and non-significant (*b* = −1.10, *SE* = 3.50, *t*(176) = −0.31, *p* = 0.76). The statistically significant interaction is consistent with attenuation of the association between the Special person score and RT differences following self-affirmation. However, given the very low reliability of Special person (α = 0.37), this result is preliminary and should not be interpreted as robust evidence of a stable facet-specific effect.

#### 3.2.2. Power Narcissism and RT Differences at 235 ms SOA

A second model examined the Power dimension at the longer SOA (235 ms), where previous exploratory analyses had suggested an association. The Condition × Power interaction was in the expected direction but did not reach conventional statistical significance (*B* = 6.90, *SE* = 3.90, *t*(176) = 1.77, *p* = 0.079, 95% CI [−0.80, 14.60]).

In the control condition, higher Power scores were not significantly associated with more negative RT difference scores at 235 ms (*b* = −7.20, *SE* = 4.10, *t*(176) = −1.76, *p* = 0.080). In the self-affirmation condition, the simple slope was significant (*b* = −14.10, *SE* = 4.00, *t*(176) = −3.53, *p* < 0.001). Because the Condition × Power interaction was not significant, this pattern does not provide confirmatory support for a differential effect of self-affirmation on the association between Power and RT responses.

#### 3.2.3. Exhibitionism

Parallel models examining Exhibitionism at both SOAs yielded no significant Condition × Exhibitionism interactions (all *ps* > 0.20). Exhibitionism was also not significantly associated with RT difference scores in either condition.

Overall, H2 received partial support. The predicted moderation effect was observed for the Special person dimension at 149 ms, but not for Power at 235 ms or Exhibitionism at either SOA.

### 3.3. Exploratory Associations Between NPI Dimensions and Self-Esteem Dimensions

To further examine the relationship between narcissism and the two self-esteem dimensions, Pearson correlations were calculated separately within each condition. These analyses were exploratory and should be interpreted cautiously given the low internal consistency of some NPI dimensions (Power α = 0.54; Exhibitionism α = 0.61; Special person α = 0.37).

#### 3.3.1. Control Condition

In the control condition, higher Special scores were associated with higher self-derogation (*r* = 0.22, *p* = 0.038) but were not significantly associated with self-competence (*r* = 0.20, *p* = 0.058). Power and Exhibitionism showed no significant associations with either self-esteem dimension (all |*r*| < 0.10, all *ps* > 0.30).

#### 3.3.2. Self-Affirmation Condition

In the self-affirmation condition, Special scores were positively associated with both self-derogation (*r* = 0.28, *p* = 0.008) and self-competence (*r* = 0.27, *p* = 0.010). Power and Exhibitionism again showed no significant associations with self-competence or self-derogation (all |*r*| < 0.18, all *ps* > 0.09).

Thus, following self-affirmation, higher Special scores were associated with both greater self-competence and greater self-derogation. However, these associations were correlational and therefore do not permit causal inferences.

These analyses provide exploratory information about associations between narcissism and self-esteem dimensions. They did not directly test whether self-competence or self-derogation predicted or moderated RT responses to negative status-related cues.

### 3.4. Exploratory Analyses by SOA (149 ms vs. 235 ms)

Finally, exploratory analyses examined whether associations between narcissism dimensions and RT difference scores varied across the two SOAs within each condition. These analyses were exploratory and were not intended to establish distinct cognitive mechanisms at the two SOAs.

#### 3.4.1. Control Condition

In the control condition, higher Special scores were associated with more negative RT difference scores at the 149 ms SOA (*r* = −0.23, *p* = 0.03) but not at the 235 ms SOA (*r* = −0.03, *p* = 0.78). Power and Exhibitionism were not significantly associated with RT difference scores at either SOA (all |*r*| < 0.12, all *ps* > 0.25).

#### 3.4.2. Self-Affirmation Condition

In the self-affirmation condition, Special scores were not significantly associated with RT difference scores at either SOA (149 ms: *r* = −0.12, *p* = 0.26; 235 ms: *r* = −0.05, *p* = 0.64). By contrast, higher Power scores were associated with more negative RT difference scores at the 235 ms SOA (*r* = −0.30, *p* = 0.004) but not at the 149 ms SOA (*r* = −0.03, *p* = 0.78). Exhibitionism showed no significant associations at either SOA (all |*r*| < 0.15, all *ps* > 0.15).

Taken together, these exploratory SOA analyses indicated that the Special dimension was primarily associated with earlier RT differences (149 ms) in the control condition, whereas the Power dimension was associated with later RT differences (235 ms) in the self-affirmation condition. These temporal patterns are consistent with the possibility that different narcissism dimensions are associated with sensitivity to negative status-related information at different stages of processing; however, the present design does not permit conclusions about underlying cognitive mechanisms.

### 3.5. Summary of Findings in Relation to the Hypotheses

H1 was supported: self-affirmation was associated with higher self-competence and lower self-derogation. H2 received limited, preliminary support. The predicted moderation effect was observed for the Special person dimension at 149 ms, but not for Power at 235 ms or Exhibitionism at either SOA. Given the low reliability of the NPI dimension scores, especially Special person, this finding requires replication using stronger facet measures.

The analyses examining associations between narcissism and self-esteem dimensions were exploratory. Although Special person narcissism was associated with self-derogation in the control condition and with both self-competence and self-derogation in the self-affirmation condition, the reported analyses did not directly test whether self-competence or self-derogation predicted or moderated RT responses to negative status-related cues.

## 4. Discussion

The present study investigated the effect of self-affirmation on self-regulation, as assessed by increased reaction time to negative cues, as well as associations among three dimensions of grandiose narcissism comprising “Power”, “Exhibitionism”, and “Special person”, and self-esteem (SE) bifactorially measured as Positive (self-competence) and Negative (self-derogation). The findings highlight some empirically meaningful distinctions between affirmed and non-affirmed groups, particularly concerning the Special and Power dimensions of narcissism (Kubarych et al., 2004), in relation to dimensions of self-esteem and self-regulation.

In the non-affirmed group, our results revealed a negative association between the Special dimension of grandiose narcissism and reaction times at the shorter stimulus onset asynchrony (SOA; 149 ms) to negative cues. Higher Special person scores were associated with faster RTs to negative cues at the 149 ms SOA in the control condition. This pattern is consistent with differential responding to negative status-related information but does not directly establish hypervigilance, automatic processing, defensive motivation, or underlying vulnerability. The Condition × Special person interaction at 149 ms should be interpreted cautiously. The Special person score had very low internal consistency (α = 0.37), so the observed association may be affected by substantial measurement error and may not represent a stable facet-specific effect. The task also does not directly measure defensive motivation.

Consistent with our previous proposals (Hardaker & Tsakanikos, 2021), we observed in the non-affirmed condition a positive association between the Special dimension of grandiose narcissism and self-derogation. Contrary to the commonly observed positive association between narcissism and SE, this finding is consistent with the possibility of greater negative self-evaluation among individuals scoring high in grandiose narcissism. The positive association between Special scores and self-derogation is consistent with the possibility that individuals who endorse stronger perceptions of personal uniqueness may also report more negative self-evaluation. This finding is compatible with, but does not by itself establish, the vulnerability proposed by the mask model. These associations between Special person narcissism and the self-esteem dimensions were exploratory. Because self-competence and self-derogation were not directly modelled as predictors or moderators of RT responses, the present study cannot draw conclusions about their associations with RT differences.

In the self-affirmation group, some pronounced shifts in the pattern of findings from the non-affirmed group were observed. Firstly, scores on Positive SE notably increased, while scores on self-derogation significantly decreased, indicating that participants in the self-affirmation condition reported higher self-competence and lower self-derogation. These findings align with existing research supporting self-affirmation’s potential to enhance adaptive self-perceptions, although the present study cannot determine whether these condition differences reflect changes in self-regulation or underlying self-views (K. Richardson et al., 2021; Zhu & Yzer, 2021).

The association observed between Special narcissism and reaction-time differences in the control condition was not observed in the self-affirmation condition. The disappearance of the association between Special scores and reaction-time differences following self-affirmation is consistent with the possibility that self-affirmation altered the processing of negative status-related information. However, because the present task does not directly measure defensive motivation, this interpretation should be regarded as provisional. The condition difference may reflect altered processing of negative status-related information; however, the present design cannot identify the underlying mechanism.

A novel positive association emerged between the Special dimension of grandiose narcissism and self-competence was observed in the self-affirmation group. This correlational association should not be interpreted as evidence that self-affirmation changed participants’ self-concept. In addition, a new association was observed between the Power dimension of narcissism and quicker reaction times to negative status-related cues at the longer SOA (235 ms). Higher Power scores were associated with faster responses at the longer SOA. This pattern may suggest that the association between Power and responses with negative status-related information is more evident at the longer temporal interval than at the shorter interval. However, the present design does not permit us to determine whether this temporal difference reflects a distinct cognitive mechanism.

In both affirmation and non-affirmation groups, the Special dimension had positive associations with both self-competence and self-derogation. The persistence of these associations highlights the complexity of narcissistic self-perception. The observed associations may reflect complexity in self-evaluative responding; however, the present data cannot establish underlying vulnerability or resistance to intervention.

This study has several notable limitations. First, the sample was predominantly female, with 163 of 180 participants being women, limiting the generalisability of the findings across genders. As the study was not designed or powered to examine gender moderation, it is not possible to determine whether the associations between narcissism, self-esteem, and reaction-time responses differ by gender. Future research should recruit more gender-balanced samples and explicitly test whether gender moderates these associations.

Although the total sample was sufficient for the planned between-condition comparison based on the a priori power analysis, the subgroup analyses involved multiple predictors, outcomes, and associations. The resulting estimates may therefore be unstable, and these exploratory findings should be interpreted cautiously. Replication in larger samples is required to assess the robustness and generalisability of these effects.

The relatively low internal consistency of the NPI dimensions, particularly Special person, should be considered when interpreting dimension-specific associations. Low reliability may attenuate associations, although it may also contribute to instability in estimates.

Finally, no direct manipulation check of self-affirmation was administered. Although the observed differences in Positive SE and Negative SE are consistent with the intended effects of the manipulation, these measures were assessed after the lexical decision task and therefore cannot independently confirm that the manipulation altered self-affirmation or self-integrity.

In conclusion, self-affirmation was associated with higher self-competence and lower self-derogation in this undergraduate sample. The observed Condition × Special person interaction at the 149 ms SOA provides preliminary evidence only, because the Special person composite had very low internal consistency (*α* = 0.37). The study cannot establish the specific mechanisms underlying the observed RT differences, including narcissistic self-regulation, defensive motivation, or nonconscious processing. Replication using psychometrically stronger measures of narcissism facets, objective prime awareness assessment, preregistered analytic plans, and approaches that address measurement errors is needed before drawing stronger theoretical or applied implications.

## Data Availability

The analyses in this study used a dataset previously published by Hardaker and Tsakanikos (2022).

## Note

1 See https://doi.org/10.6084/m9.figshare.33973261 for examples of the self-affirmation paradigm preselection sheet and scrambled sentence task and the lexical decision task word/non-word lists.
